# A survey of “mental hardiness” and “mental toughness” in professional male football players

**DOI:** 10.1186/2045-709X-22-17

**Published:** 2014-04-15

**Authors:** Rainer Wieser, Haymo Thiel

**Affiliations:** 1 C3 Chiropractic Clinic, 223-225 Pantbach Road, Rhiwbina, Cardiff, Wales CF14 6AE, UK; 2 Anglo-European College of Chiropractic, 13-15 Parkwood Road, Bournemouth, Dorset BH5 2DF, UK

**Keywords:** Mental toughness, Mental hardiness, Survey, Male professional football players

## Abstract

**Background:**

It is not uncommon for chiropractors to be associated with sports teams for injury prevention, treatment, or performance enhancement. There is increasing acceptance of the importance of sports psychology in the overall management of athletes. Recent findings indicate mental hardiness can be determined reliably using specific self-assessment questionnaires. This study set out to investigate the hardiness scores of professional footballers and examine the correlation between two questionnaires. It also included a mental hardiness rating of players by two coaches, and examined differences in hardiness and mental toughness between national and international players.

**Methods:**

Two self-assessment questionnaires (modified Sports Mental Toughness Questionnaire [SMTQ-M] and Psychological Performance Inventory [PPI-A]) were completed by 20 male professional footballers. Two coaches, independently rated each player. A percentage score from each questionnaire was awarded each player and an average score was calculated ({SMTQ-M % + PPI-A %} ÷ 2). The PPI-A and SMTQ-M scores obtained for each player were analysed for correlation with Pearson’s correlation coefficient. Cohen’s kappa inter-reliability coefficient was used to determine agreement between coaches, and between the players’ hardiness scores and coaches’ ratings. The independent t-test was used to examine differences between national and international players.

**Results:**

The players’ scores obtained from PPI-A and SMTQ-M correlated well (r = 0.709, p < 0.001). The coaches ratings showed significant, weak to moderate agreement (Cohen's kappa = 0.33). No significant agreement was found between player self-assessments and coaches’ ratings.

The average ({SMTQ-M % + PPI-A %} ÷ 2) mean score was 77% (SD = 7.98) with international players scoring 7.4% (p = 0.04) higher than non-international players.

**Conclusions:**

The questionnaires (SMTQ-M and PPI-A) correlated well in their outcome scores. These findings suggest that coaches moderately agree when assessing the level of mental hardiness of football players. There was no agreement between player self-assessment and ratings by coaches. Footballers who play or had played for national teams achieved slightly higher mental hardiness scores.

Either questionnaire can offer the clinician a cost-effective, valuable measure of an individual’s psychological attributes, which could be relevant within the wider context of bio-psycho-social model of care.

## Background

Optimal sports performance depends on physical and psychological components. Often, millimeters and micro-seconds are decisive factors between success or failure. When athletes, especially top performers of similar physical ability, have reached their optimal physical limits, the only difference for their success could depend on psychological components. One assumes that the competitor with greater control over his or her mind will emerge as the victor. In professional football over recent years, expectations of coaches and players have increased dramatically. Ever-growing demands to win, the fear of failure, and targets which are often set too high, are common causes to lead to psychological stress. However, it appears that some players or coaches are able to handle difficult and stressful situations better than others. Do they have certain characteristics or mental attributes which enable them to cope better with or even thrive under stress, and how can on measure these mental features?

Consequently, a deeper understanding of the contribution of certain elements of sports psychology in the overall treatment and management of athletes can be very beneficial for physiotherapists, chiropractors and other health care professionals who deal with sports injury management. One of the authors (RW) had been working with the local championship football team as a sports chiropractor for the past seven years, and during this period had come across the concept of “mental hardiness” and its possible relevance within the context of sports performance.

The concepts of mental hardiness [1,2] and mental toughness [3,4] are closely linked to the construct of what has been termed “positive psychology” by Seligman and Csikszentmihalyi [5]. A central tenet of the positive psychology paradigm is that stressors and other inordinate demands are inherent to the human condition. However, the paradigm also assumes that they can be sources of strength, through which adversity can be endured and even transcended. Physical, emotional, and social stressors can further personal development and enhance mental strength in some individuals. Such people are often able to tap into previously unknown capacities, perspectives, and virtues [6].

The concept of “hardiness” was first introduced by Kobasa [1] by trying to find a link between stressful life events and the onset of illness. The study reported on two groups of individuals who faced similar stressful life events, one group became ill after their stressful experience whilst the other remained healthy. It was proposed that the reason why individuals reacted differently to similar stressful events was a personality difference, best described as hardiness. The research found that the individuals who remained healthy showed more hardiness than the individuals who fell ill. The personality construct of hardiness emerged from existential psychology: a viewpoint that proposes that meaning in life is created through the decisions people make. It is composed of the “three C’s - Commitment, Control and Challenge” [2]. Commitment is the predisposition to be involved with people and events, rather than to be isolated and detached [7,8]. Control is the belief that one can influence outcomes, rather than feeling powerless to change anything. Finally, Challenge is the attitude that change is not only inevitable, but beneficial, and that it offers incentives to learn and develop, rather than serving as a threat. Research conducted at the U.S. Military Academy at West Point found that mental hardiness protected Army reserve personnel mobilised for the Persian Gulf War [9]. In this study, the higher the hardiness level, the greater the ability of soldiers to experience life- and combat-related stress without apparent negative health consequences, such as post-traumatic stress disorder or depression.

The most important skills and techniques applied in contemporary sports psychology are: imagery, goal setting, self-talk, stress management, team building, efficacy management, attention control, emotion regulation and mental toughness [10]. A winning mentality and desire have been identified as key attributes of mentally tough soccer players in addition to other previously reported qualities such as self-belief, physical toughness, work ethic/ motivation, and resilience [11]. Key cognitions reported by mentally tough soccer players enable them to remain focused and competitive during training and matches, which reflects that positive self-talk can help some players in dealing with challenging situations [6].

Sheard and colleagues [4] identified mental toughness as a crucial attribute for success in sport. There are seven components to mental toughness, such as confidence, constancy, control, determination, self-belief, positive cognition and visualisation. These components can be measured with self-assessment questionnaires such as the Sports Mental Toughness Questionnaire (SMTQ) and the Psychological Performance Inventory (PPI-A) [12,13]. More specifically, the SMTQ measures confidence, constancy and control, whereas he PPI-A measures determination, self-belief, positive cognition and visualisation. Both questionnaires have been tried on larger sample sizes of athletes [4,14]. In answer to the question, “does a screening test accurately detect a hardiness score?” previous research has established that these tests are reliable and consistent instruments for self-assessment [14,15]. A cross-national analysis of mental toughness and hardiness in elite university rugby league teams revealed that Australian University players had significantly higher mean scores on positive cognition, visualisation, mental toughness and challenge than their opponents from Great Britain [13]. It has also been shown that individuals with greater hardiness scores are less likely to report injuries [2], and that players with higher mental toughness have “greater pain tolerance and therefore under-report injury and are often not compliant with their treatment” [16].

The first aim of the present study was to investigate the hardiness scores of professional footballers and examine the correlation between the outcome scores of a slightly modified SMTQ (SMTQ-M) and the PPI-A. As previous research has been primarily based on self-assessment scores provided by athletes only, the second aim of this study was to ask two coaches to independently assign a hardiness score to each player, and then to examine for the level of agreement between the coaches’ and players’ scores, and also between the two coaches themselves.

## Methods

### Study design

This study was a quantitative survey based on numerically derived data scores from two established psychology questionnaires adapted and slightly modified for professional footballers.

### Ethical approval

Ethics approval was obtained through the Research and Ethics Sub-Committee of the Anglo-European College of Chiropractic, Bournemouth, United Kingdom.

### Recruitment of subjects

The subjects were a convenience sample of football players. This consisted of 20 players of a local professional Welsh football team. RW had been the chiropractor to the football team for more than seven years.

### Setting

The data collection took place at the training grounds of Cardiff City Football Club, South Glamorgan, Wales.

### Data collection - players

A short player profile data collection tool captured details on a player’s nationality, age, years of professional experience, highest level of performance and criteria for reporting an injury. The SMTQ and the PPI-A were slightly modified by removing numbers and letters across the options and replacing them with tick boxes so as not to influence the choice of response. A further alteration was made to the SMTQ, in that question one and five were made identical. This was done to assess for consistency of responses. The players were given an information sheet about the study and asked to complete a consent form. RW was on site to give further clarification on how to complete the questionnaires if required.

### Data collection - coaches

Two experienced coaches, who had been with the club for more than five years were asked to independently rate all of the players for mental hardiness using the following scoring key: above average = 1, average = 2, below average = 3.

### Data analysis

As the questionnaires were based on different scales, their overall numerical scores were converted into percentage scores to allow comparison between data sets. The average percentage score for each footballer ({SMTQ-M% + PPI-A%} ÷ 2) and a mean score for the whole sample of players was established. Pearson’s correlation coefficient was used to determine the strength of linear dependence between the SMTQ-M and the PPI-A scores. Cohen’s kappa inter-reliability coefficient was used to determine the level of agreement between the two football coaches. In order to assess the level of agreement between the players’ self-ratings with the coaches’ ratings, each individual player’s score was converted into categorical data to be in line with the coaches scoring key. The independent t-test was used to examine differences in the average percentage scores between national and international players. PASW Statistics (version 18) software for apple mac computers was used for data analysis.

## Results

The footballers’ ages ranged from 18 to 33 years (mean = 24.8 years, SD = 4.63), and the length of their professional experience from one to 15 years (mean = 7.7 years, SD = 4.84). Eleven players (55%) responded that they report injury immediately after it had occurred, whilst six indicated that they report injury when they experience mild pain and three when they experience severe pain only. Three players were currently injured and were therefore not playing at the time of data collection. Six players had been playing for less than one year for the football club, 11 players for one to five years, and three players for more than five years. Eight players either had been or were members of a national team, two players had played in the Premier League in the past, and 10 played at Championship level. Two players were of foreign origin, and English was therefore their second language.

**Figure 1:**
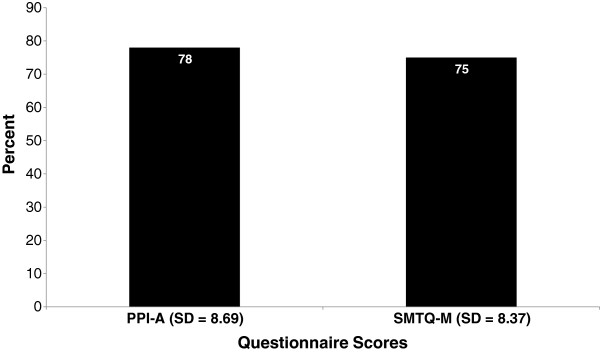
Mean group percentage scores for PPI-A and SMTQ-M.

Figure 1 shows the mean group percentage scores of both questionnaires (PPI-A and SMTQ-M). There was a slightly overall higher score on the PPI-A questionnaire, with a mean of 78% (SD = 8.69) compared to the SMTQ-M with a mean of 75% (SD = 8.37), but this difference was not statistically significant. There was a significant difference (independent t-test, p = 0.04) in the average mean score between international and non-international players, with the international players scoring 7.4% higher (4.7% - 14.4%; 95% CI). The sub-group of eight international players achieved an average mean score of 81% (SD = 4.88) compared to the overall mean score of the 12 Non-Internationals, who had a lower mean score of 74% (SD = 8.41).

**Figure 2:**
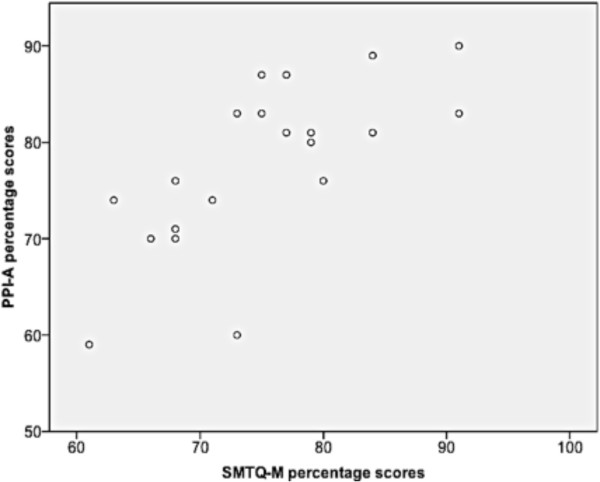
Scatter plot between SMTQ-M and PPI-A (r = 0.709, p <0.001).

The Pearson's correlation revealed that there was a significant linear relationship between the SMTQ-M and the PPI-A scores (r = 0.709, p < 0.001) (Figure 2). Inter-reliability between the two coaches when rating the players was weak to moderate (k = 0.33), whilst there was no agreement between the coaches’ ratings and the average player self-ratings (Coach A: k = -0.17, Coach B: k = -0.07).

**Figure 3:**
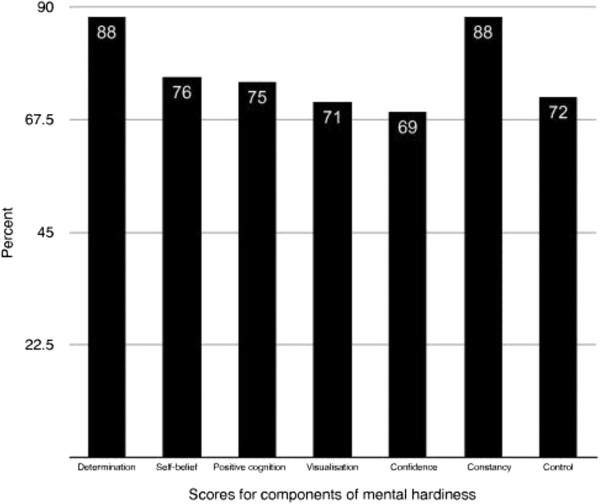
The teams mean percentage scores of the 7 hardiness components.

Comparison of the sample’s mean percentage scores for the seven hardiness components revealed high scores for control and determination, with confidence and visualisation achieving the lowest scores (Figure 3). The analysis of the team’s mean percentage scores of the 7 hardiness components revealed that at the time of the data collection the team scored well on the mean determination (88%, SD = 10.11) and constancy (88%, SD = 8.81) scores. However, the team’s mean confidence (69%, SD = 12.36), self-belief (76%, SD = 10.24) and positive cognition (75%, SD = 9.30) scores were relatively low. The mean visualisation score was 71% (SD = 16.50) and the mean control score 72% (SD = 13.83).

## Discussion

In contrast to most studies, which have focused on academy players, this study researched mental hardiness in professional footballers. An interesting finding of this study is that 45% of professional football players will report injury when they experience some form of pain, 30% will report when they experience mild pain, with 15% only reporting severe pain. One of the most likely reasons for being reluctant in reporting injuries could be a player’s fear of losing his place in the first team. Delayed reporting makes it difficult for the team doctor and physiotherapist to deal with injuries effectively. Late diagnosis and delayed monitoring of a possible injury increases the chance of re-injury and could lead to more severe and chronic injuries. A self-centered mindset of a mentally tough performer could therefore be a disadvantage to the whole football team. The interest of the team should always take priority over personal achievement.

Some of the other findings in this study, such as the correlation between SMTQ-M and PPI-A scores, the statistically significant higher hardiness scores of international football players concur with previous research [14,17]. However, to our best knowledge no previous study has utilised the two questionnaires in a combined fashion in order to arrive at one numerical score for mental hardiness. We feel that this approach provides for a good overall indicator of mental hardiness or toughness, as it combines most of the sub-components previously referred to within the literature.

Previous work had also found that athletes performing at an international level score higher in relation to mental hardiness [4,14]. Similar findings were demonstrated in this study, with the sub-group of eight international players achieving an average mean score which was 7% above that of non-internationals.

The analysis of the team’s mean percentage scores of the seven hardiness components revealed that at the time of the data collection the team scored well on the mean determination score and constancy. However, the team’s mean confidence, self-belief and positive cognition scores were relatively low and would therefore indicate room for improvement. The mean visualisation score was also low and as only certain players use this technique, there would be no need to address this issue for the benefit of the team.

This study also showed that there was a strong significant linear correlation between the SMTQ-M and the PPI-A questionnaires, which concurs with previous research by Sheard [11,15]. A recent paper by Crust and Swann [17] measured the correlation between the SMTQ and the Mental Toughness Questionnaire (MTQ 48) on a sample of 110 male athletes and also found that there was a significant positive correlation in higher order mental toughness scores (r = 0.75; p < 0.001). This compares well to the findings of the present study and shows that these tools produce consistent results even when slightly modified.

The ratings of the players by the two coaches showed weak to moderate agreement (Cohen's kappa = 0.33) and no agreement was found between player self-assessments and the ratings given by the coaches. These findings agree with those of another study [18] which also investigated the inter-rater agreement of two coaches, who took repetitive measurements on 21 professional academy players using two different questionnaires. This suggests that even amongst professional football coaches, there are considerable differences in interpreting the behaviours and characteristics of players and that their judgments are subjective. Individual coaches are likely to have their own interpretation of mental toughness as a common an agreed definition does not really exist [19]. Similarly the present study reflected the findings of previous work [18,20] in that there was no significant agreement between the coaches’ scores and the player self-assessment scores. However, even though the results showed little agreement between the individual coaches, and between the coaches’ and the player self-assessment scores, the scores provided for a good platform for player feedback and making individuals more self-aware of their various traits in relation to mental hardiness. The mean score was used as an indicator for overall team spirit. With this additional information, further options were now available to the coaches and players to either address weak areas, focus on strong psychological sub-components, or both, overall aiming for a more holistic player development. These outcomes could be very useful, as long as players are willing to adapt their behaviour, and coaches are willing to address and develop these components within their coaching methods.

### Limitations of study and suggestions for further research

The data were collected on the basis of responses made at a single point in time. As such the results could have been strongly influenced by how the players were feeling at the time, especially as they had lost a number of preceding games. It was essential that the players answered the questions honestly, they may have over-estimated their abilities in their responses. Some played at several levels in different leagues during their career. However, the player’s questionnaire only took into account the highest level of play throughout their career, even if they were not at this highest level currently. Although the sample represented a relatively homogenous group of professional footballers, it was of a small size and as such its findings cannot be generalised to other football teams. Furthermore, there is a risk of Type 1 and Type 2 errors. Although the coaches had been instructed to complete their questionnaires independently from each other, the researcher had no control over this to ensure that it actually happened. When the coaches considered a player they may have sub-consciously included in their assessment different components such as talent, skill and past performance.

Future research could include a more phenomenological approach to personality research. More longitudinal research into mental hardiness in contexts outside sports performance is required. Another interesting investigation could determine how intellectual skills influence the outcome scores on surveys, as it is possible that people with a better educational background will achieve higher hardiness scores.

## Conclusion

Personality is a mixture of genetic predisposition and environmental influences. Individual traits and the environment can act as co-determinants of behaviour. Personality structure involves both a stable core of attitudes, values and beliefs about self, which remain relatively unchanged after early childhood. Exploring these characteristics through self and external assessment can be a valuable experience for a footballer, even if at present, there is no evidence upon which one can directly link mental hardiness with success in football.

The present study could only explore some components of the construct and was focused on the quantitative measurement of mental hardiness. There was good correlation between the two questionnaires and both are equally sufficient to measure characteristics of mental hardiness. The advantage of combining the questionnaires to achieve an overall mean score may lie in providing a more comprehensive assessment of an individual’s mental hardiness. However, if due to practical or time saving reasons, one chooses to use only one of the questionnaires, the authors recommend the PPI-A as it measures slightly more components compared to the SMTQ-M.

The results showed that even amongst elite level professional soccer coaches, there is little agreement when judging the mental hardiness of their players and the players themselves have a different self-judgment compared with the coaches’ ratings. However, through enhanced self-knowledge and feedback via the coach one could conceivably address certain weaknesses and build on strengths.

The understanding of the concept of mental hardiness is still evolving and the complexities of human psychology and its nature are such that it is difficult to condense them easily into discrete sub-components. However, in the present study the combination of these sub-components appeared to be a good indicator of an overall sense of the “team spirit”.

“Knowing others is clever. Knowing yourself is enlightenment. Controlling others requires force. Controlling yourself requires strength” (Lao Tzu).

## Competing interests

The authors declare that they have no competing interests.

## Authors’ contributions

RW: Concept and design of the study, collection and entry of data, analysis and interpretation of the manuscript, final approval of the manuscript. HT: Concept and design of the study, drafting and revising the manuscript and final approval of the manuscript. Both authors read and approved the final manuscript.
